# Semaglutide for Treating Obesity Induced by Craniopharyngioma Resection: A Successful Case Study

**DOI:** 10.1210/jcemcr/luad074

**Published:** 2023-07-27

**Authors:** Cristina Sciacovelli, Ginevra Moschione, Silvia Garelli, Uberto Pagotto

**Affiliations:** Division of Endocrinology and Diabetes Prevention and Care, IRCCS Azienda Ospedaliero-Universitaria di Bologna, 40138 Bologna, Italy; Department of Medical and Surgical Sciences (DIMEC), Alma Mater Studiorum University of Bologna, 40138 Bologna, Italy; Division of Endocrinology and Diabetes Prevention and Care, IRCCS Azienda Ospedaliero-Universitaria di Bologna, 40138 Bologna, Italy; Department of Medical and Surgical Sciences (DIMEC), Alma Mater Studiorum University of Bologna, 40138 Bologna, Italy; Division of Endocrinology and Diabetes Prevention and Care, IRCCS Azienda Ospedaliero-Universitaria di Bologna, 40138 Bologna, Italy; Division of Endocrinology and Diabetes Prevention and Care, IRCCS Azienda Ospedaliero-Universitaria di Bologna, 40138 Bologna, Italy; Department of Medical and Surgical Sciences (DIMEC), Alma Mater Studiorum University of Bologna, 40138 Bologna, Italy

**Keywords:** hypothalamic obesity, neoplasm, semaglutide, body weight, pharmacological therapy

## Abstract

Surgical treatment of craniopharyngioma often leads to a rapid and dramatic weight gain, leading to hypothalamic obesity. Treatment focused on the diet, physical activity, and different types of drugs have very often provided unsatisfactory results. To date, no data have been reported on hypothalamic obesity (HO) regarding the use of semaglutide, a novel type 1 receptor glucagon-like peptide-1 agonist, to limit body weight gain after surgical removal of a neoplasm, despite its already documented efficacy in obesity treatment. In this case report, we tested semaglutide in an 18-year-old patient with HO induced by a surgical intervention for craniopharyngioma. A very favorable treatment response was found in terms of body weight reduction and improvement in metabolic parameters. Our patient lost more than than 30 kg after only 6 months of therapy, which has never been reported before in the literature on HO.

## Introduction

Hypothalamic obesity (HO) is a common sequel of mediobasal hypothalamic insult due to brain tumors, neurosurgical intervention, or radiotherapy. Surgical management of craniopharyngioma induces HO when the tumor removal involves posteriori hypothalamic structures including the mammillary bodies. There are several factors underlying the increase in morbid long-term body weight, such as alterations in food control mechanisms, decreased energy expenditure, and impairment of the autonomic nervous system. In addition, circadian rhythm disorder, sleep quality reduction, visual dysfunction, and neurological sequelae may further increase the level of obesity [1].

HO is very often unresponsive to traditional treatments. In fact, lifestyle modifications with adherence to a hypocaloric diet and regular physical exercise are insufficiently effective. Pharmacological treatment has also been highly disappointing, although many approaches have been considered in order to bypass damaged hypothalamic pathways. Lastly, although bariatric surgery could help to tackle HO, its results are often disappointing, since weight loss is generally short-lived [1], probably because the hypothalamic integrity is necessary to maintain a sustained long-term weight loss [2].

Glucagon-like peptide-1 agonist (GLP-1) receptor agonist (GLP-1 RA), initially used in the treatment of type 2 diabetes mellitus, represents a successful novel pharmacological paradigm to treat simple obesity [3].

GLP-1 is a gut-derived incretin hormone, whose receptors are found in the gastrointestinal system and in various areas of the central nervous system [3, 4]. This hormone increases the secretion of glucose-dependent insulin and reduces the secretion of glucagon and the rate of gastric emptying [1, 3, 4]. GLP-1 binds to receptors in the vagus and to the appetite-related sites in the hindbrain (nucleus of the solitary tract and area postrema), the hypothalamus (arcuate and dorsomedial nuclei), and the limbic system (nucleus accumbens) among many others. It thus acts as a satiety hormone, promoting reduced food intake and modulating appetite- and reward-related brain areas [3-5]. In addition to the hypothalamus, other areas of the brain may be involved in the GLP-1 signals of reduced food intake. Thus, GLP-1 message is transduced through redundant neuronal pathways and not only at the level of the hypothalamus, which is usually damaged in HO. This could be one of the reasons why GLP-1 RAs work efficiently on HO.

There are few studies on GLP-1 RA in patients with HO. Of these, exenatide and liraglutide have been tested in patients with HO, with variable results related to weight loss and improvement in metabolic parameters [4, 5].

Here we present the case of a young boy with severe obesity that developed after surgical intervention for craniopharyngioma who responded strongly to semaglutide in terms of body weight and metabolic improvement.

## Case Presentation

A 16-year-old male underwent a neurological examination for the progressive onset of generalized asthenia, hypersomnia, mild dysphagia, slurred speech, memory impairment, recurrent headache, and worsening visual disturbance with difficulty in visualizing moving objects. He had lost 20 kg in the previous 6 months following a reduction in caloric intake due to the sensation of early gastric fullness. All these symptoms were responsible for significant limitations in daily activities and poor performance at school.

## Diagnostic Assessment

**Figure 1.**
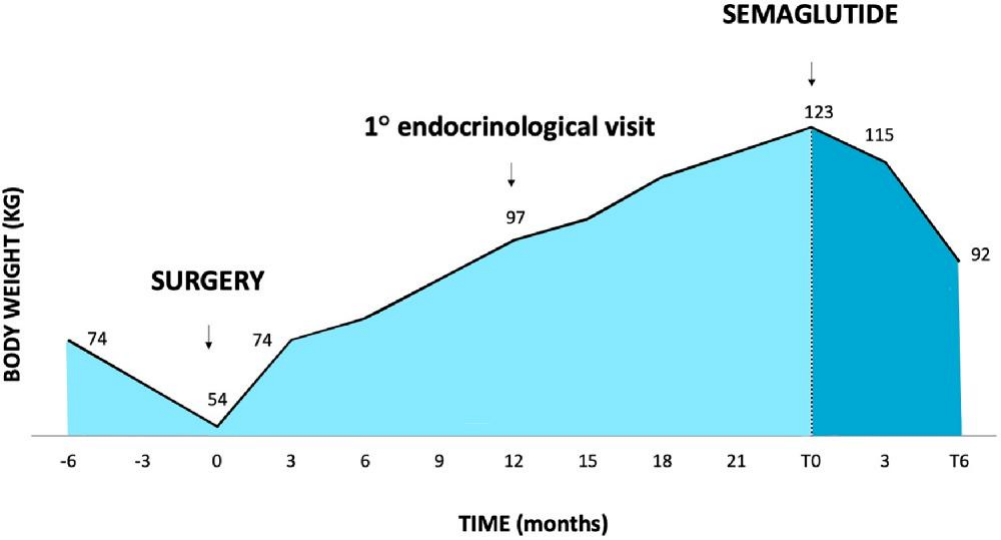
Change in body weight after transsphenoidal surgery, before semaglutide administration (T0) and after 6 months of therapy (T6).

**Figure 2.**
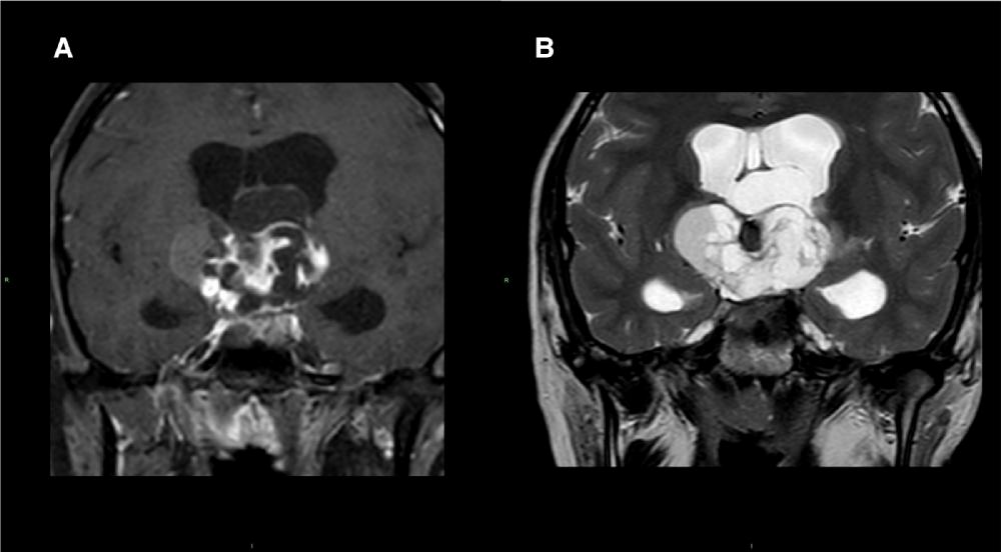
(a) and (b): Coronal T2 and enhanced T1-weighted images [figures (a) and (b), respectively] demonstrate a large expansive formation (5 × 4 cm) with irregular enhancement and associated cystic components in the median suprasellar region, with expansion to the third ventricle imprinting the chiasma region, obliterating the interventricular foramina of monroe, with dilatation of the ventricular system in the supratentorial site and caudally to the prepontine cistern (not shown). The lesion is iso-hyperintense in T1-weighted and hyperintense in T2-weighted images.

At physical examination, the patient weighed 54 kg with a body mass index (BMI) of 21.1 kg/m2 (57.5th percentile) (Fig. 1). Neurological examination showed a mild left-sided weakness. Ophthalmic evaluation revealed an incomplete right homonymous hemianopia and bilateral atrophy of the temporal edge of the optic disc. Blood tests were performed with evidence of hypogonadotropic hypogonadism, GH deficiency, and a slight increase in prolactin, while hypothalamic-pituitary-adrenal axis and thyroid tests showed normal results. Brain magnetic resonance imaging with gadolinium contrast showed a large expansive formation (5 × 4 cm), consistent with a craniopharyngioma (Fig. 2a and 2b).

In July 2020 the patient underwent transsphenoidal surgery to remove the tumor. The histological examination described an adamantinomatous craniopharyngioma. He developed post-surgical panhypopituitarism and central diabetes insipidus, thus supplementation for hormone deficiencies was initiated.

Three months after surgery, the patient reported significant changes in symptoms, with a significant increase in appetite. He had regained his premorbid weight (74 kg, BMI 28.9 kg/m2, 96.6th percentile) (Fig. 1) and was thus referred for a nutritional evaluation.

## Treatment

One year after the surgery, the patient came to the attention of our endocrinological center due to a weight problem, reporting difficulty in following the low-calorie diet prescribed by the nutritionist, as well as inertia in relation to physical activity.

At the first examination in July 2021, the patient weighed 97 kg (Fig. 1) with a BMI of 37 kg/m^2^. Blood tests were performed with evidence of a mild hyperinsulinemia (fasting glucose was 65 mg/dL, 3.61 mmol/L; insulin was 21.8 mU/mL, 151.4 pmol/L). A low glycemic index diet combined with physical activity were prescribed, with a monthly nutritional follow-up. Meanwhile the patient was also followed by a psychologist due to suspected eating disorder behavior.

Despite adequate hormonal replacement therapy and repeated dietary and lifestyle counseling, the patient continued to gain weight, complaining of persistent hunger with binge eating on many occasions. Metformin 1 g twice a day was prescribed. Follow-up brain magnetic resonance imaging showed a stable neuroradiological picture, without signs of recurrence.

In July 2022, 2 years after surgery, the patient's weight was 123 kg (+69 kg from presurgery weight) (Fig. 1), with a BMI of 46.9 kg/m2, and blood tests showed severe and worsening hyperinsulinemia (Table 1). Off-label treatment with semaglutide was thus combined with metformin after acquiring the parent's informed consent, with a 12-week titration regimen (initial treatment at 0.5 mg/week increasing by 0.5 mg every 4 weeks until a dose of 2 mg/week was reached).

**Table 1.** Laboratory results before and 6 months after semaglutide therapy

Parameters	Before semaglutide administration	6 months after semaglutide therapy*^a^*
Weight (kg)	123	92
BMI (kg/m^2^)	46.9	35.1
Glucose mg/dL (mmol/L)	58 (3.22)	65 (3.61)
Insulin µU/mL) (pmol/L)	39.4 (273.63)	5.8 (40.28)
HOMA IR Index	5.16	0.93
HbA1c % (mmol/mol)	5.44 (36)	4.53 (26)
LDL cholesterol mg/dL (mmol/L)	154 (3.99)	132 (3.42)
HDL cholesterol mg/dL (mmol/L)	38 (0.98)	40 (1.04)
Triglycerides mg/dL (mmol/L)	203 (2.29)	98 (1.11)
ALT U/L µkat/L	31 (0.52)	23 (0.38)
AST U/L µkat/L	40 (0.67)	17 (0.28)
Creatinine mg/dL (µmol/L)	0.86 (76.2)	0.96 (84.86)
Hemoglobin g/dL (g/L)	15.6 (156)	16.8 (168)
Albumin g/dL (g/L)	—	4.48 (44.8)
Prealbumin mg/dL (mg/L)	—	25.5 (255)
Sodium mmol/L	137	139
Potassium mmol/L	3.8	4.3
FT4 pg/mL (pmol/L)	6.6 (84.95)	13.9 (178.91)
Testosterone ng/mL (nmol/L)	2.15 (7.46)	5.71 (19.81)
IGF-1 ng/mL (nmol/L)	238 (31.18)	193 (25.28)

Abbreviations: ALT, alanine transaminase; AST, aspartate transaminase; BMI, body mass index; FT4, thyroxine free; HDL, high-density lipoprotein; HbA1c, hemoglobin A1c; HOMA IR, Homeostasis Model Assessment for insulin resistance; LDL, low-density lipoprotein.

a Hormonal replacement therapy was not changed since the beginning of treatment with semaglutide and metformin, except for levothyroxine titrated to weight. Hydrocortisone modified release 25 mg/d, desmopressin 150 mg/d, somatotropin 0.2 mg/d, testosterone undecanoate 1000 mg 1 vial (intramuscular injection) every 10 weeks, at present levothyroxine 175 mcg 1 day/weekly and levothyroxine 150 mcg 6 day/weekly. The hormonal variations observed during the 6-month treatment period were not significant enough to account for the patient's weight changes, nor did they have a clinically negative impact.

## Outcome and Follow-up

Soon after the first weeks of the low-dose treatment, the patient reported a normalization of appetite and a sensation of satiety that he had not felt for the previous 2 years. After only 6 weeks, he had already lost 8 kg and continued to lose weight over the next few weeks (Fig. 1). The patient also no longer reported episodes of losing control with food, which has ceased to be his obsession. No adverse events were reported, except for occasional nausea but without impacting daily life.

Before starting the treatment and 6 months afterwards, the patient was screened for eating disorders using the Binge Eating Scale (BES) and Three-Factor Eating Questionnaire. The BES includes 16 items and examines both behavioral manifestations (eating large amounts of foods) and sensation/cognition during a binge episode (loss of control, guilt, fear of being unable to stop eating). The Three-Factor Eating Questionnaire includes 51 items aggregated into 3 main scales: restrained eating, disinhibition, and hunger. Restrained eating measures the amount of intentional restraint in food intake; disinhibition measures the loss of control in eating patterns and social/emotional eating; hunger measures the subjective feeling of hunger [6] (Table 2). While a diagnosis of binge eating disorder according to the BES was likely before treatment, after treatment it was completely excluded (Table 2). Blood tests were repeated, with evidence of improvement in dyslipidemia and insulin resistance, in the absence of signs of malnutrition (Table 1).

**Table 2.** Questionnaire results before and 6 months after semaglutide

Questionnaire	Before semaglutide	6 months after semaglutide
Binge eating disorder*^a^*	39	9
Cognitive restraint of eating*^b^*	10	6
Dishinibited/uncontrolled eating*^c^*	14	6
Hunger*^d^*	13	1

a Binge Eating Disorder Scale: < 17 improbable, 17-27 possible, > 27 probable.

b Three-Factor Eating Questionnaire: > 11 indicative of restraint.

c Three-Factor Eating Questionnaire > 8 indicative of disinhibition.

d Three-Factor Eating Questionnaire > 7 indicative of hunger.

Together with weight loss the patient felt more inclined to do physical activity, with better performance, and experienced an overall significant improvement in mood and quality of life. After 6 months of treatment, he reached a weight of 92 kg, BMI of 35.1 kg/m2 (−31 kg from the beginning of the treatment) (Fig. 1). At present he is still taking 2.0 mg/week semaglutide.

## Discussion

To the best of our knowledge, this is the first case of semaglutide administration to tackle HO after craniopharyngioma surgical treatment. Semaglutide 2.4 mg/weekly was recently approved by the Food and Drug Administration for adults with obesity or overweight [7]. However, in Italy, where our study was carried out, this drug is only available for diabetic patients (at 1 mg/weekly), hence the option for *off-label* treatment.

Semaglutide is known to also potently reduce weight and BMI in adolescents between 12 and 18 years with primary obesity [8], ie, a similar age to the patient in our case study. Semaglutide differs from other GLP-1 RAs in its longer half-life and because it leads to a greater weight loss. It also seems to activate different areas of the central nervous system compared to liraglutide [9]. Studies on mice have shown that it regulates weight loss by direct interaction with diverse GLP-1 receptor populations and by directly and indirectly affecting the neural pathways involved in food intake, reward, and energy expenditure, such as the parabrachial nucleus [9]. Pharmacokinetics might also explain the more evident weight loss attributed to semaglutide compared with other GLP-1 agonists.

Semaglutide seems to reduce craving and regulate food preference, which suggests that it may affect food intake via hedonic as well as homeostatic pathways and therefore could be used for reducing the hyperphagia typical of patients with HO. This was the case in our patient who went from a clinical picture suggestive of a binge eating disorder to obtaining an excellent control of hunger and a good perception of satiety [9, 10].

The more than 10 BMI point reduction in a 6-month treatment timeframe with semaglutide described here, although obtained in a single case report, represents a unique exception in the literature. In fact, in the series of 9 patients treated with GLP-1 analogues with daily administration (1 liraglutide, 8 exenatide), none of them were able to reach a similar extent of body weight loss [4]. Similarly, in the double-blind placebo-controlled randomized 36-week study of the effect of exenatide 2 mg on body weight in HO (ECHO trial), in which 42 patients were enrolled, the percentage difference in BMI change was not significant in comparison to the placebo, although a heterogenous response was shown among the patients. Some of the patients who underwent exenatide treatment showed a significant reduction in body weight and waist circumference, although lower than the values reached in our single case [5].

The effect of semaglutide on reducing basal insulin hypersecretion as shown in our patient is particularly important. Hyperinsulinism is a classical feature of HO that derives from the hypothalamic disinhibition of the vagal output. The increased stimulated level of insulin may contribute to maintaining obesity by promoting energy storage in the adipocyte, thus resulting in increased fat mass. In an attempt to reduce hyperinsulinism, many different drugs have been tested over the years, with modest results [1].

Larger studies are needed to fully evaluate the efficacy of semaglutide in HO. However, we believe that the promising results obtained in this single case report may pave the way to stimulate ad hoc trials on future novel GLP-1 agonists aimed at such a difficult-to-treat form of obesity. If treatment with novel GLP-1 RA agonists is successful, it could also be important to understand whether starting antiobesity therapy as soon as possible after surgical intervention in patients with hypothalamic dysfunction would help in limiting the onset of severe obesity.

## Learning Points

- To date, treatment for HO has proven to be poorly effective.
- Semaglutide, which acts on several sites of the central nervous system and not only at the hypothalamic level, could be beneficial in treating the compulsive aspects of eating behavior in HO.

## Data Availability

Data sharing is not applicable to this article as no datasets were generated or analyzed during the current study.
